# “Human Flourishing with Dignity”: A Meta-Ethnography of the Meaning of Gardens for Elderly in Nursing Homes and Residential Care Settings

**DOI:** 10.1177/23333936211035743

**Published:** 2021-07-31

**Authors:** Inger-Lise Magnussen, Johanne Alteren, Terese Bondas

**Affiliations:** 1 Nord University, Stokmarknes, Norway; 2 Molde University College, Norway; 3 University of Stavanger, Norway

**Keywords:** residents, nursing home, residential care, garden, meta-ethnography, nursing care and dignity

## Abstract

This study aims to identify and synthesize qualitative research regarding residents’ experiences of gardens while living in nursing homes and residential care facilities. To provide an optimal nursing environment inspired by nature, we need to derive knowledge from the residents’ perspective. An interpretive meta-synthesis approach, a meta-ethnography, was chosen for this study. Altogether, six articles representing three continents and comprising 124 participants were included. The six articles that fulfilled the inclusion criteria were analyzed and synthesized according to Noblit & Hare’s seven phases of meta-ethnography and the recent eMERGe guidelines. Four themes were identified: (1) The garden—a place to feel a connection with life, (2) the garden—a place to sense and find comfort, (3) the garden—a place to feel healthy and alive, and (4) the garden—a place to relate past and present. An overarching metaphor, “human flourishing with dignity,” offers a deeper understanding of the meaning of the garden for older people in nursing homes and residential care. This meta-ethnography provides a reflective, systematic, data-driven synthesis based on literature spanning ten years. Rather than simply relying on retelling, the narration of experiences according to the primary researcher’s descriptions and interpretations results in new knowledge. The significance of gardens for older people’s health and well-being needs to be given greater attention and space in nursing practice, education, and health policies.

## Introduction

Nature, landscapes, and gardens have promoted restoration and well-being (Winterbottom & Wagenfeld, 2015). Notably, Florence Nightingale (1860) brought nature into nursing when she wrote about its healing powers in relation to nursing care. In health care literature, gardens are defined in various ways, such as sensory gardens (Winterbottom & Wagenfeld, 2015), wander gardens (McCaffrey et al., 2011), therapeutic gardens (Detweiler et al., 2012), and forest gardens (Corazon et al., 2019). Despite different concepts and target groups, the promotion of health and well-being serves as a commonality. In this study, a garden is described as a delimited and facilitated outdoor area with a safe environment, where plants and trees can stimulate senses such as sight, hearing, smell, taste, and touch (Berentsen et al., 2008). The goal of gardens in nursing homes and residential care is to alleviate suffering, promote health, and provide freedom and access to fresh air and natural surroundings for the residents (Berentsen et al., 2008; Winterbottom & Wagenfeld, 2015). The residents in nursing homes and residential care facilities are in this study also described as “older people.” The garden may thus offer comfort and inspiration, thereby motivating and facilitating both active and passive participation. Therefore, it could be included as an individual part of nursing care in residential care facilities for older people. In this study we will identify experiences of gardens from the perspectives of older people living in nursing homes and residential facilities.

## Background

### Nature and Garden—A Place for Health and Interaction

Previous research highlights that both visual and physical access to outdoor spaces are important for older people to experience freedom and movement (Orr et al., 2016), including those with dementia (Van Hecke et al., 2018). Natural landscapes and gardens have a well-known therapeutic potential (Motealleh et al., 2019) and are core features of a healing environment that ensures older people’s emotional (Blake & Mitchell, 2016), mental (Corazon et al., 2019), physical, social, psychological, and cognitive health and well-being (Uwajeh et al., 2019; Whear et al., 2014). Older people living at home considered gardening meaningful, which strengthens the sense of belonging to nature (Leaver & Wiseman, 2016), and integral to who they are as a person (Cheng & Pegg, 2016). For those with dementia, the garden became “the heart” of their lives (Marsh et al., 2018, p. 176). In hospitals, the environment plays a critical role in the patient’s experience of well-being, health, and support, especially on the advent of serious illness (Blaschke, 2017). Even historical landscapes and urban parks seem to improve mental health, well-being (Heaslip et al., 2020), and aging processes (Wang & Rodiek, 2019).

The nature-human interaction facilitated a sense of coping with illness and normalcy (Gagliardi & Piccinini, 2019), and the interaction between older people with dementia, staff, and relatives provided a sense of joy, community, belonging, and personalization (Whear et al., 2014). Besides, it keeps older people connected with the world (Cooley et al., 2020), with others, and their roots and preserves their culture and traditions (Wang & Glicksman, 2013). From the employees’ perspective, the residents prefer spontaneous visits to the garden as opposed to planned ones (Liao et al., 2020). Moreover, the residents’ family members appreciated the garden’s restorative effect (Bengtsson & Carlsson, 2013). The benefits elicited by older people depend on the caregivers’ attitudes towards the use of nature and gardens (Sion et al., 2020), and since the systematic use of gardens does not appear as part of individual nursing, changes are needed (Cooley et al., 2020).

### Gardens in Residential Care Facilities

Gardens and gardening helps the elderly to create an attractive home environment both in their own homes (Scott et al., 2015), and in nursing homes and residential facilities (Roy et al., 2018). Gardens are especially important for nursing home residents, and to promote health and well-being, the proximity and access to nature may determine older people’s choice of residence (Freeman et al., 2019). Relocation may cause great upheavals and interruptions in older people’s relationships with nature and gardens, and the value of owning a home is culturally rooted (Vasara, 2015). Hence, gardens ought to be considered as a crucial part of a nursing home or residential care setting to provide and facilitate access and participation (Kamioka et al., 2014). Older people, who appreciate gardening, value the view and access to gardens (Detweiler et al., 2012), which prevents feelings of isolation, control, and discomfort for people with dementia as well (Fisher et al., 2018).

In the Netherlands, meaningful and tailored outdoor activities in nursing homes seemed to reduce older people’s experiences of boredom (Sion et al., 2020); in Australia, older people described gardening as a passion. Furthermore, in New Zealand, gardening became a way to age gracefully (Boyes, 2013), and a positive correlation between gardening, health, and wellbeing was determined (de Bell et al., 2020). The individual, adapted, and customized environments and activities of nursing homes and residential care settings may encourage both active and passive participation (Goto et al., 2017). Also, the location and size of the garden impact its use, and small- and medium-sized gardens seem more predictable and safer than large gardens (Shi et al., 2019).

Previous research on gardens concerning health and well-being promotion predominantly targeted the design, localization, and access to the garden. Our previous research had a professional perspective (Magnussen et al., 2019). However, a key issue related to gardens in nursing care is the older people’s experiences regarding their relationship to and their use of the garden at residential facilities. To the best of our knowledge, no meta-synthesis has focused on older peoples’ experiences with gardens in nursing homes and residential care facilities. We find it important to emphasize the older peoples’ voice and knowledge since it concerns their life, home, and health.

### Health Geography

The relationship with nature is imperative for human existence, and older people tend to have well-established relationships with nature and gardens (Andrews, 2006, 2016). The theory of health geography focuses on the dynamics of place-health, where places, people, life, and experience of health are related to each other (Andrews, 2006), also in nursing contexts (Andrews, 2002). The place is a complex cultural and symbolic phenomenon created through the relationship between humans and their surroundings (Andrews & Moon, 2005). The garden is often associated with domestic surroundings and home. Residents and relatives believe that access to the garden should be an integral part of life in a nursing home (Cooney, 2012). Moreover, maintaining interests and activities is important in establishing relationships with new places and finding a home to avoid the feeling of homelessness and rootlessness (Molony, 2010). The theory of health geography may shed light on the garden’s relational aspect and significance for safeguarding older people’s longing for contact with nature (Andrews, 2014).

### Aim

The aim of this meta-ethnography was to identify and synthesize qualitative studies that describe and illuminate residents’ experiences with gardens while living in nursing homes and residential care environments. The goal is to enlarge the knowledge base from the residents’ perspectives and enhance the present use and planning of gardens in nursing homes while seamlessly integrating them in nursing care to improve health and well-being for older people.

## Methods

### Design

Meta-ethnography was chosen for this study as an interpretative meta-synthesis approach (Noblit & Hare, 1988) that intended to move beyond the original qualitative studies of a phenomenon (Bondas & Hall, 2007) by synthesizing the individual study findings (France et al., 2019). Meta-ethnography can lead to a new conceptual understanding of any phenomenon by systematic analysis and comparing and contrasting translations of findings in the original studies (Noblit & Hare, 1988). The seven phases of meta-ethnography by Noblit and Hare guided the study as a nonlinear interpretative approach. Besides, we adopted the eMERGe guidelines recently developed by France et al. (2019) to improve the completeness and clarity of meta-ethnographic reporting.

### Data Collection and Analyses

#### Phase 1 getting started

Our interest in the topic was guided by questions on the international perspectives of residents in nursing homes that had arisen from our previous research collaboration: action research on sensory gardens in a Norwegian nursing home, from staff and leader perspectives (Magnussen et al., 2019). When reviewing the literature, as part of our previous research on the professional perspectives, it was evident that the perspectives of older persons needed more attention. The current pandemic situation was an important motive for the study.

#### Phase 2 deciding what is relevant

In phase 2, we decided to focus on the residents’ perspectives regarding the gardens that have been created, especially for use in relation to nursing homes and similar residential housing, including dementia care. We worked on developing the inclusion and exclusion criteria through the initial process of finding relevant studies, partly known from our previous studies. The criteria were decided in a back-and-forth process in a team effort when studies were retrieved. One example of this process was discussions on the meaning of the type of garden and the final decision for inclusion and exclusion.

The following inclusion criteria were used in the selection of studies:

- Older people, aged 65 years and over, living in nursing homes or residential care settings (i.e., care dwellings, rest homes, and other care facilities for older people) that comprised gardens
- Peer-reviewed original qualitative research studies according to the aim published in scientific journals. Disciplinary restrictions were not applied
- Date of publication: 2010 to 2020
- Mixed methods if the qualitative findings were clear
- Mixed participants if the voices of the older people were clear
- English and Scandinavian languages

Exclusion criteria:

- Older people opting for home care nursing, day care centers, hospitals, or short rehabilitation programs when the garden was not part of the residential facility
- Indoor gardens
- General studies concerning experiences of nature, public gardens, parks, and farms
- Quantitative studies

**Figure 1.**
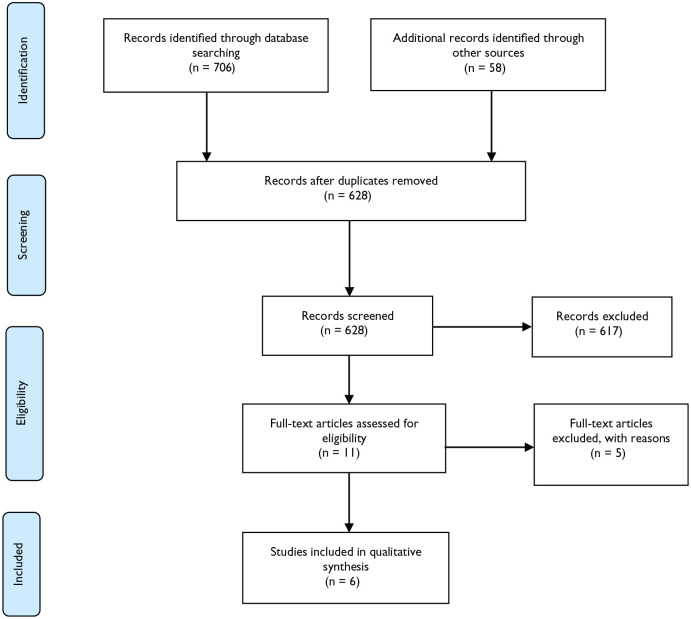
Flow-chart of the literature search.

The first author conducted the search process with identification and screening, and discussions with the other authors contributed to clarification and agreement throughout the study process. The search strategy aimed at evaluating all the relevant articles. First, we checked the reference lists of our previous research and performed database searches (CINAHL, ProQuest, PubMed, Wiley, and Scopus) employing the terms “sensory garden OR therapeutic garden OR garden AND old people AND dementia NOT child AND qualitative NOT quantitative AND nursing home OR residential care.” We applied limitations according to the databases, such as older people aged 65+ and 80+ years. Reference searches from the new articles and author searches continued, as well as checking journal manuals and titles and abstracts in relevant international journals: *Alzheimer’s and Dementia, Global Qualitative Nursing Research, Health and Place, International Journal of Older People Nursing, Journal of Advanced Nursing, Journal of Clinical Nursing, Journal of The Housing for the Elderly*, and the Nordic journals: *Hoitotiede, Klinisk Sygepleje*, and *Sykepleien Forskning*. Lastly, we checked Google Scholar, Oria, and Web of Science for any possible hits. We also used the supplied searches in the databases for similar articles, which are included in the total number of hits. The flow chart (PRISMA) (Moher et al., 2009) is depicted in Figure 1.

We distinguished 764 records through database searching and other sources. After the removal of duplicates (using EndNote and manual procedures), 628 records were screened via the title, keywords, and abstract. We excluded 617 records consisting of reviews, posters, news, and conference abstracts and those with a focus on rehabilitation, hospitals, home care, indoor gardens, or children. All the studies were assessed by two authors, and a consensus was attained concerning their inclusion in the next phase (i.e., critical appraisal). We appraised the 11 full-text articles for eligibility and qualification according to the Critical Appraisal Skills Programme (CASP) criteria for qualitative studies (http://www.casp-uk.net/checklists) (Table 1). CASP analysis determined the final six records to be included in the synthesis. Only a few disagreements between the reviewers were discussed in the meetings and resolved. Moreover, the studies had sufficient information to ensure proper interpretation that may contribute to new knowledge (Noblit & Hare, 1988).

**Table 1.** Critical Appraisal of the Included Articles (CASP).

Critical appraisal questions										
Article	1	2	3	4	5	6	7	8	9	10
Lo et al. (2019)	Y	Y	Y	Y	Y	Y	Y	Y	Y	Y
Reynolds (2016)	Y	Y	Y	Y	Y	Y	Y	Y	Y	Y
Dahlkvist et al. (2020)	Y	Y	Y	Y	Y	Y	Y	Y	Y	Y
Johansen and Gonzalez (2018)	Y	Y	Y	Y	Y	N	Y	Y	Y	Y
Tsai et al. (2020)	Y	Y	Y	Y	Y	Y	Y	C	Y	Y
Raske (2010)	Y	Y	Y	Y	Y	Y	C	N	Y	Y

*Note.* Critical appraisal questions: (1) was there a clear statement of the aims of the research? (2) is a qualitative methodology appropriate? (3) was the research design appropriate to address the aims of the research? (4) was the recruitment strategy appropriate to the aims of the research? (5) was the data collected in a way that addressed the research issue? (6) has the relationship between researcher and participants been adequately considered? (7) have ethical issues been taken into consideration? (8) was the data analysis sufficiently rigorous? (9) Is there a clear statement of findings? and (10) how valuable is the research?. Y = yes, N = no, C = can’t tell.

Table 2 specifies the characteristics of the included studies after an initial data extraction, which are process of the author, year, country, aim, method, participant characteristics, and main findings.

**Table 2.** The Characteristics of the Included Studies.

Author/year	Country	Aim	Method	Participant characteristics	Main findings
Lo et al. (2019)	China	The aim was to explore the perceptions and experiences of a group of frail and/or pre-frail older nursing home residents in relation to horticultural therapy	Qualitative descriptive approach. Interviews Thematic analyzes (Braun & Clarke, 2006)	22 residents in nursing home F: 14 M: 8 Mean age: 86.3 years Frail or pre-frail state, with normal or mildly impaired cognition	Horticultural therapy was an enjoyable and pleasant activity, and a good pastime for the residents. Horticultural activities facilitated socialization among the residents and engaged them physically and mentally by giving purpose and meaning to their lives. The program was hardly mentioned by the staff outside the sessions. The residents’ perceived quality of life in the institution, which they now call “home”
Reynolds (2016)	US	To better understand the contradiction of how use of gardens despite perceived benefits, this study explores how individuals living in elder care environments perceive the value of nature and personal factors that may influence use of garden spaces	Individual and focus-group interviews (Strauss, 1990)	32 older adults in residential care facilities: 10 independent living 17 assisted living 5 personal care F: 24 M: 8 Mean age: ND Intact memory and able to communicate	The views of nature are of fundamentally importance for the resident’s well-being. Access to nature influences their choice of facility, and the use of garden spaces is influenced by the way in which individuals prefer to enjoy nature. Humans have an inherent need of connect with life and the processes in nature, and this fundamental need must not be ignored when designing residential care facilities, where the residents are able to independently access their community and outdoor environments
Dahlkvist et al. (2020)	Sweden	To describe the gardens and their use by individuals living at residential care facilities with high rating on restorative values	A descriptive design with behavior mapping, observations, and field notes (Gifford, 2016) Content analysis (Patton, 2015)	11 residents in residential care facilities F: 8 M: 3 Mean age: 86 years Residents with or without cognitive impairment	The gardens were mainly used for socialization and relaxation and stimulated the residents’ senses and evoked memories from the past. These restorative values were interpreted as a sense of being away and fascination, and not having opportunities for outdoor visits gave feelings of disappointment and reduced well-being
Johansen and Gonzalez (2018)	Norway	The objective of this study was to explore and describe what characterizes experiences and memories linked to nature and the outdoor environment in a Norwegian context for residents staying permanently in a nursing home	Qualitative exploratory and descriptive design Semi-structured interviews Analysis: Systematic text condensation (Malterud, 2012)	8 nursing home residents F: 3 M: 5 Age: 62–90 years Residents with ability to narrate stories from their own life	Being in contact with nature promoted engagement among the residents, and they wanted changes, sensory impressions, and experiences. The residents became fascinated in nature and experienced thriving, well-being and actively engaged in others. The nature helped the residents to recall contact with their own roots, identity, integrity, and life history, and to remember. In old age the nature was used differently than before, and changes occurred based on the patient’s health condition
Tsai et al. (2020)	Australia	This research explores the current understanding of aged-care gardens and highlights the lack of homemaking in aged-care outdoor space design Two questions about the residents’ experiences of the landscape in aged-care facilities were asked	Phenomenological framework, unstructured interviews, Go-Along video-recording and digital storytelling Content analysis with thematic approach (Boyatzis, 1998)	35 residents in residential care facilities with no significant dementia or severe illness F: ND M: ND Age: ND 5 staff	The residents are not merely passive users of gardens, they are active creators, shaping their outdoor environment through gardening and creating meanings in their local landscape that contribute to their experience of being “home.” The garden had a positive impact on residents’ quality of life, especially in terms of meaningful daily activities, enjoyment of daily life, relationship with others, and functioning as independently as one would like. The enabling garden had also a positive impact on the quality of life for staff and volunteers
Raske (2010)	USA	To conduct an in-depth evaluation of the impact of the construction and use of an enabling garden on resident quality of life in a rural nursing home	Qualitative, exploratory, and descriptive design Interview Content analysis (Patton,1990)	16 residents, 4 with dementia, in residential care facilities F: 10 M: 6 Mean age: 81.4 years 15 Staff 6 Family members 6 garden volunteers	The garden had positive effects on resident quality of life, particularly in terms of meaningful daily activities, enjoyment of daily life, relationships, and functional competency. The residents appreciated being involved in the garden-design, they loved free access to the garden, it gave them their freedom back, they found garden activities meaningful and pleasant, which evoked good memories and feelings. Gaining access to garden was important when relocated in nursing home and residential care facilities

*Note.* Table 2 Author and year of publication, country, aim, method, participants characteristics, and main findings.

#### Phase 3

Reading the included studies in full text, each of the authors made notes while reading and discussing the preliminary perceptions. The authors were well-acquainted with the research disciplines of nursing and health sciences in the included studies, and the theoretical and professional variation mitigated the overlooking of details.

#### Phase 4

We determined the relation between the studies after several readings. The authors made an initial assumption about the relationship being analogous or reciprocal between the included studies (Noblit & Hare, 1988) and independently performed the data extraction procedures in pairs by listing the findings using line-by-line coding (France et al., 2019). Particularly, line-by-line coding for the translation process was chosen to derive findings for the analysis that were narratively described in the text of the primary authors, in addition to the themes.

#### Phase 5

The interpretation of the studies allowed a comparison of the findings from one study to another. This process was not linear; instead, we went back and forth between the findings and the primary studies. This phase was also performed in pairs, and the discussion resulted in consensus. In Table 3, the translation process is exemplified, where the article in the first column is the index paper that had rich findings (France et al., 2019). The translation process involved treating the findings as analogies (Noblit & Hare, 1988), enabling comparison between similar findings between the studies. An analogy is not literal, word-for-word, but idiomatic, that is, translating the meaning of the findings.

**Table 3.** Examples of Translation of the Findings of the Studies.

Lo et al. (2019)	Reynolds (2016)	Dahlkvist et al. (2020)	Johansen and Gonzalez (2018)	Tsai et al. (2020)	Raske (2010)	Codes
1.1. HC is an enjoyable fun activity	2.1. Sense of adventure	3.1. Sense of being away and fascination and just have fun	4.1. Wanting Change Fascination		6.1. Enjoyment in resident lives	Adventure Fascination Being away Enjoyable fun
1.2. HC is a pastime filling their boring day with purpose and meaningfulness	2.2. Sense of accomplishment when participating in outdoor activities	3.2. Weeding garden beds Digging in the growing beds, some raking	4.2. Active and engaged	5.2. Daily routines, sense of ownership and control, picked flowers, watering and moved plants—daily ritual 5.17. Capacity and ability to actively changing the SL	6.2. Provided R with meaningful activities, selecting and planting seeds, having meals or picnics in the G Love activities	Meaningful and engaged pastime Coping, create and complete Breaks of routines and a boring everyday Bright spot in everyday life
1.7. Happiness with HC and more positive to life	2.7. N essential to their well-being	3.7. Relaxation, decreased well-being if not access	4.7. Well-being Thriving 4.13. Attitude to N changes	5.7. Making a nice G make my life happier Comfortable exercising	6.5. Seating in the G is comfortable Love the feeling of being out Glad to come out	Happiness Feelings of well-being and comfort Thriving Positive attitude to life

*Note.* R = resident; G = garden; HC = horticultural therapy; SL = surrounding landscape; N = nature.

#### Phase 6

Synthesization of translations denoted the analyses of the translations, thus moving beyond the findings of the individual studies to the second level of synthesis (Bondas & Hall, 2007; France et al., 2019; Noblit & Hare, 1988). Based on the translations, themes were generated, and we consulted the primary studies for validity in addition to several discussions that were held before the attainment of consensus. Refutations were not found. A line of argument synthesis (Noblit & Hare, 1988), as a metaphoric model, was finally created through an in-depth creative, interpretative, and systematic back-and-forth analytic process. In a team effort, we moved between the themes and the translations of findings, going back to the included studies for understanding. The synthesis provides an in-depth understanding of the meaning of gardens in nursing homes and residential care facilities from older people’s perspectives.

## Results

The six included articles represent three continents, namely, Europe, America, and Asia, for the period between 2010 and 2020. The overview of the studies is exhibited in Table 2. The total number of participants was 124, and since the study by Tsai et al. (2020) did not account for gender, the distribution for the remaining five studies was 30 men and 59 women. Information about the average age was specified in three of the studies, while two were missing this information. Furthermore, the study by Johansen et al. (2018) described an age range of 62 to 90 years. Concerning the location of the gardens, they were integrated as part of the nursing home’s outdoor environment or located nearby, and their characteristics coincide with this study’s description of a garden. The descriptions in the included studies of the type of garden, garden design and size, concept, and mobility aids are shown in Table 4.

**Table 4.** Overview of Type of Gardens.

Author/year/country	Type of garden	Garden design and size	Concept	Mobility aids
Lo et al. (2019)/China	Horticultural therapy garden (HCG)	Principles of HCG are (a) plants which are easy to procure, (b) flexibility in conducting the sessions, (c) plants that show a sense of continuity, (d) participation adapted to the resident’s capability and needs	Garden	ND
		Garden size: ND		
Reynolds (2016)/US	Institutional garden associated with residential care facilities with proximity to gardens	Two residential settings with three different gardens, relatively proximity to each other:	Garden	Six residents used cane or walker, four used wheelchair and 10 used none
		1. Assisted-living facility surrounded by landscaped beds, a small vegetables garden, and a fenced lawn with walking path Garden size: 60 feet wide by 44 feet deep		
		2. Independent-living villas with either their own lawn or patio gardens Garden size: 78 feet wide by 66 feet deep		
		3. A common garden for both living facilities, covered by landscaping space, water feature and trees Garden size: 62 feet wide by 42 feet deep		
Dahlkvist et al. (2020)/Sweden	Institutional garden	Maps of the gardens shows lawn, green spaces, trees, beds, paths, fences, and access to the garden (doors)	Garden	Walk by themselves or using wheelchair
		Garden design and size: ND		
Johansen and Gonzalez (2018)/Norway	Outdoors surroundings and nature	Rural surroundings	Outdoor environment	ND
Tsai et al. (2020)/Australia	Landscape associated with residential care facilities	Vegetated landscape areas that include communal spaces and space designated for use by individual units	Garden landscape	ND
		Garden size: ND		
Raske (2010)/USA	Nursing home garden	The garden was constructed in the central courtyard, with automatic doors that allowed free access, and was in its second year	Enabling garden	ND
		Garden size: ND		

*Note.* Tabel 4 Overview of type of garden, garden design and size, concepts, and mobility aids. ND = not described.

The design of the studies included descriptive, exploratory, grounded theory, and phenomenological approaches. Data collection was conducted using individual or focus group interviews, except Dahlkvist’s et al. (2020) study, which used behavior mapping, field notes, and conversations. All studies relied on textual analyses, such as content or thematic analysis and text condensation.

Through the translation process (phase 6) of the included studies, four main themes and subthemes emerged. Finally, a synthesis was presented as a metaphoric model.

### The Garden—A Place to Feel a Connection With Life

The first theme, the garden—a place to feel a connection with life, constitutes three subthemes: (a) Connecting with oneself, (b) Connecting with others, and (c) Connecting with nature.

#### (a) Connecting with oneself

Gardens and nature were experienced as places that create and maintain personality, identity, and dignity (Johansen & Gonzalez, 2018; Raske, 2010; Reynolds, 2016; Tsai et al., 2020). Gardens reflected a deeply personal history and became a way of connecting to life in general (Tsai et al., 2020), and nature as a historical legacy provided a coherence of self-identity whereby the boundaries of the time were blurred between the past and present self (Reynolds, 2016). Nature was closely linked to the residents’ identity, as one of them said: “If you were born by the ocean, then you feel it’s something that lies in your blood” (Johansen & Gonzalez, 2018, p. 9). The natural surroundings helped the residents find their place and discover themselves (Johansen & Gonzalez, 2018), and one 86-year-old resident stated, “the garden gives me the feeling that my dignity is respected” (Raske, 2010, p. 345).

#### (b) Connecting with others

The garden became an arena for making friends and experiencing togetherness, in addition to creating joyful and inclusive social communities (Dahlkvist et al., 2020; Johansen & Gonzalez, 2018; Lo et al., 2019; Raske, 2010; Reynolds, 2016; Tsai et al., 2020). The interaction between the residents in the garden occurred spontaneously (Johansen & Gonzalez, 2018) or was organized by the nurses (Reynolds, 2016), and this interaction developed social relationships (Tsai et al., 2020) and awareness. Notably, a 69-year-old resident stated, “He picks a bag of cherry tomatoes for me to eat” (Raske, 2010, p. 343). Gaining friends and engaging in conversations resulted in socialization, but in a joyful and meaningful way, while working together provided happiness (Lo et al., 2019; Tsai et al., 2020) and prevented loneliness (Dahlkvist et al., 2020). When outdoors, the residents were talking (Johansen & Gonzalez, 2018), viewing (Lo et al., 2019), and sharing meals (Dahlkvist et al., 2020) and flowers with each other (Tsai et al., 2020) in an inclusive way.

#### (c) Connecting with nature

Being connected with nature is cardinal in old age when relocated to a nursing home or residential care facility (Tsai et al., 2020). The relocation initializes a new stage of life, and the connection with nature and people was described as a symbiotic relationship (Reynolds, 2016; Tsai et al., 2020). The garden kept the mind working, and one 99-year-old female resident stated, “I love the feeling when I’m out in the garden. It takes me back to my childhood and prior gardening” (Raske, 2010, p. 344). The course of nature became a depiction of their life course: “It was green and lush with flowers, but when autumn comes, they wither one by one (said in a sad tone of voice)” (Johansen & Gonzalez, 2018, p. 10). The presence of nature created an affinity for plants, and residents were engaged in plant life processes during the seasons (Reynolds, 2016). Also, outdoor activities tend to preserve the residents’ ties to nature (Reynolds, 2016).

### The Garden—A Place to Sense and Find Comfort

This second theme, the garden—a place to sense and find comfort, is accentuated by two subthemes: (a) Adds color and taste to life, and (b) Seeking the tranquility of nature.

#### (a) Adds color and taste to life

Sensory and esthetic impressions, both outdoors and from the inside (Dahlkvist et al., 2020; Johansen & Gonzalez, 2018; Lo et al., 2019; Raske, 2010; Reynolds, 2016; Tsai et al., 2020), were greatly appreciated by the residents, and one of them said, “It is good, just watching it grow, seeing it alive and changing every day, is truly amazing” (Lo et al., 2019, p. 1233). The residents cherished watching the roads and seeing children play (Dahlkvist et al., 2020), and became fascinated with the fragrance of flowers and the sounds of butterflies and hummingbirds (Dahlkvist et al., 2020; Lo et al., 2019; Raske, 2010; Reynolds, 2016). Further, they were reported to be captivated by the pretty colors and shapes of the plants and trees (Johansen & Gonzalez, 2018; Lo et al., 2019; Raske, 2010). The beauty of nature nourished the resident’s senses, such as through the smell of the lilacs (Dahlkvist et al., 2020) or the chirping of the birds and tasting of fresh tomatoes (Raske, 2010). Some of the residents added a personal touch to the garden by making the environment more beautiful and esthetically pleasing (Tsai et al., 2020). Generally, the main reason for outdoor visits was their longing for the sunny weather and fresh air, although the weather was no obstacle to being outdoors (Dahlkvist et al., 2020; Johansen & Gonzalez, 2018).

#### (b) Seeking the tranquility of nature

The residents found the garden to be a place of self-discovery, peace (Raske, 2010; Tsai et al., 2020), and well-being (Johansen & Gonzalez, 2018; Lo et al., 2019). The garden turned out to affect residents’ spiritual well-being, and some of the residents reported interactions with the garden as pleasant, peaceful, comfortable, and an escape from the noise inside (Raske, 2010; Reynolds, 2016; Tsai et al., 2020). These feelings were connected with flourishment (Johansen & Gonzalez, 2018) and relaxation (Dahlkvist et al., 2020). Moreover, being outdoors represented a wonderful sense of freedom (Johansen & Gonzalez, 2018; Tsai et al., 2020), and there was a resounding “Yes” to questions pertaining to the meaning of being in nature (Reynolds, 2016, p. 305). Contrariwise, when the opportunity to go outside was absent, the residents experienced decreased well-being, as one of them stated, “If I can’t be outside and get some sun I become hysterical” (Dahlkvist et al., 2020, p. 7). The garden offered serenity and tranquility (Raske, 2010; Tsai et al., 2020), and this atmosphere contributed to a more positive attitude towards life (Lo et al., 2019; Tsai et al., 2020) and nature (Johansen & Gonzalez, 2018).

### The Garden—A Place to Feel Healthy and Alive

The third theme, the garden—a place to feel healthy and alive, comprises two subthemes: (a) Bringing joy and life into everyday life and (b) Human roots anchored in the landscape.

#### (a) Bringing joy and life into everyday life

Being out in the garden among joyous and fascinated residents is alluring (Dahlkvist et al., 2020; Johansen & Gonzalez, 2018; Lo et al., 2019; Raske, 2010; Reynolds, 2016). One of the residents experienced a sense of adventure and accomplishment when participating in outdoor activities (Reynolds, 2016, p. 304), and another found the horticultural activities to be nice and funny (Lo et al., 2019, p. 1234). Besides, one participant revealed the process of gifting as enjoyable (Tsai et al., 2020, p. 5), and others yearned to get out for physical functionality (Raske, 2010, p. 344). For the older people, connecting with animals, especially dogs and cats, became a means of socialization (Dahlkvist et al., 2020), and watching weather conditions and seasonal changes inspired conversations (Dahlkvist et al., 2020; Lo et al., 2019; Reynolds, 2016). In springtime, the residents felt more alive, and they seemed to actively take part to ignite this “spark of life” (Johansen & Gonzalez, 2018, p. 8). Just being outdoors provided an enjoyable feeling of being away (Dahlkvist et al., 2020; Johansen & Gonzalez, 2018); however, the significance of nature changed in old age, and some of the residents demonstrated a conciliatory attitude (Johansen & Gonzalez, 2018). For those with compromised physical status, the garden became even more important (Lo et al., 2019).

#### (b) Human roots anchored in the landscape

The sensation of being one with nature was defined by the residents as being rooted (Johansen & Gonzalez, 2018) or actively participating in the landscape (Tsai et al., 2020). Getting involved in designing the outdoor area became a way of personalization and meaningful engagement (Dahlkvist et al., 2020; Raske, 2010; Tsai et al., 2020); a married couple stated, “. . . when we first arrived, it was pretty untidy. My husband didn’t like to see it . . . he cleaned it all up . . . putting plants, shrubs . . .” (Tsai et al., 2020, p. 4). Some of the residents preferred indoor activities and perceived the outdoors from the inside (Johansen & Gonzalez, 2018; Reynolds, 2016). Nature and gardens were attributed a particularly profound and sentimental value (Raske, 2010; Reynolds, 2016), as they provided a sense of home and ownership (Raske, 2010). Nonetheless, a few residents longed for the landscape that reminded them of their childhood (Dahlkvist et al., 2020; Johansen & Gonzalez, 2018; Raske, 2010). Access to gardens and nature often influenced the choice of residence (Dahlkvist et al., 2020; Reynolds, 2016; Tsai et al., 2020), and a 79-year-old resident said that he could not leave after the enabling garden was installed. He said, “no place else has a garden” (Raske, 2010, p. 347).

### The Garden—A Place to Relate Past and Present

The fourth theme, the garden—a place to relate past and present, is elaborated by two subthemes: (a) Those were the days—personal memories and (b) Garden—an escape from everyday life.

#### (a) Those were the days—personal memories

Nature and gardens aided residents to reminisce individual life histories and memories, and the staff preserved and created new experiences together with and about the residents (Dahlkvist et al., 2020; Johansen & Gonzalez, 2018; Raske, 2010; Reynolds, 2016; Tsai et al., 2020). For some of the residents, nature served as a historical legacy of meaningful persons and events (Reynolds, 2016). Plants from family and friends evoked memories, and the garden became a harbor of strong memories of home, childhood, and other relevant places (Tsai et al., 2020). As expressed by a female resident, “In Sidney, I had this magnificent garden . . . it was a memory garden in the sense that I have memories of the people” (Tsai et al., 2020, p. 5). Memories became an important part of the garden and represented a significant garden experience that was highly valued as a source of life contentment (Raske, 2010) and meaning (Johansen & Gonzalez, 2018; Reynolds, 2016; Tsai et al., 2020).

#### (b) Garden—an escape from everyday life

Activities in the garden provided meaningful and engaging pastimes for most residents and offered welcoming breaks from monotonous routines and boring days (Dahlkvist et al., 2020; Johansen & Gonzalez, 2018; Lo et al., 2019; Raske, 2010; Reynolds, 2016; Tsai et al., 2020), although some preferred that their routines, such as bathing schedule, remain undisrupted (Lo et al., 2019). Specifically, daily routines in the garden evoked a sense of ownership and control over the outdoor space (Tsai et al., 2020), and sometimes they could also bend some rules (Raske, 2010; Tsai et al., 2020). Taking part in weeding or digging in the growing beds, raking (Dahlkvist et al., 2020), and selecting and planting seeds (Raske, 2010) gave the residents a sense of agency (Tsai et al., 2020). Furthermore, gardening helped to revise old lessons (Tsai et al., 2020), strengthened skills, and coping abilities (Dahlkvist et al., 2020), while promoting sharing and acquisition of knowledge (Raske, 2010). As one resident clarified, “I used to be a plant killer . . . but I learned new skills . . . I can grow some now” (Lo et al., 2019, p. 1233). The gardens provided a patient-friendly environment (Dahlkvist et al., 2020) where the residents could freely wander without the possibility of getting lost (Raske, 2010). Reduced mobility, obstacles, and the absence of accompaniment could pose a threat to the residents’ sense of security (Dahlkvist et al., 2020; Lo et al., 2019; Raske, 2010; Reynolds, 2016). One of the residents got a horrible feeling when she had to push the walker on the grass, while another felt sad and afraid of getting lost while walking alone in the garden (Dahlkvist et al., 2020). The residents regarded the staff and family as being too busy to talk to them about the garden (Lo et al., 2019), but some of the managers encouraged them to participate and take responsibility in maintaining a nice garden area (Tsai et al., 2020), which fostered mutual respect and generosity (Raske, 2010).

### The Synthesis

**Figure 2.**
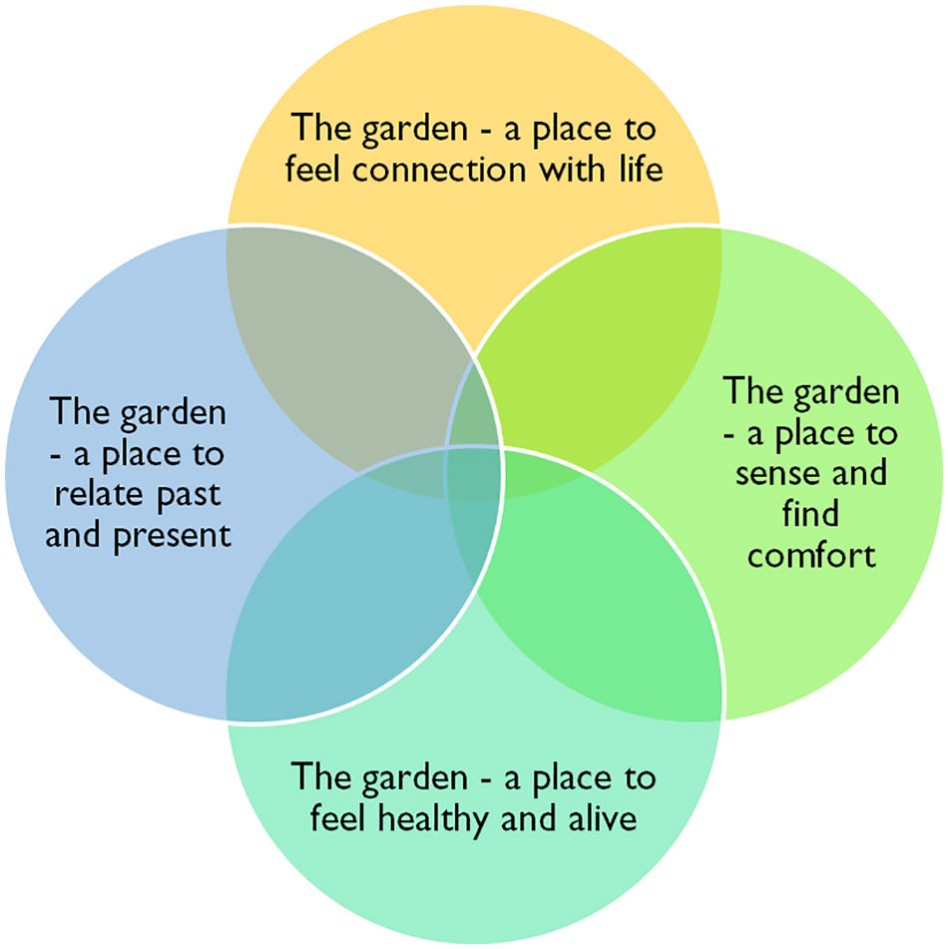
A metaphorical model: Flower “human flourishing with dignity.”

The garden in nursing homes and residential care environments is a place to feel a connection with life, to sense and find comfort, to feel healthy and alive, and to relate the past and present. From these findings, the metaphor “Human flourishing with dignity” emerged. The wholeness of human beings is illustrated as a flower, and all petals must be intact to be whole, as illustrated in Figure 2. In this metaphor, flourishing means thriving, meaningful activities, healing, and being accepted as a person, and it has an impact on physical and mental health and the development of social fellowship. The metaphor “Human flourishing with dignity” symbolizes the vital role of nature and gardens in nursing homes and residential care—from the older people’s perspective. Just as the flower is nourished by the soil, older people, even those suffering from dementia, are nurtured by their meaningful past experiences (Marsh et al., 2018). Similarly, a plant can be moved and rooted elsewhere if some of the roots are taken care of, and the same applies to people. The metaphor brings the findings one step further and reveals some of the human soil required for older people to grow and flourish with dignity when relocated to nursing homes and residential care facilities. This human soil, which includes the resident’s life history and relationships with nature and people, needs to be cared for respectfully.

## Discussion

In this study, we created a synthesis of the experiences of gardens for older people living in nursing homes and residential care settings. The synthesis “Human flourishing with dignity” is based on the notion that gardens are a place to feel a connection with life, with oneself, with others, and with nature. Similarly, the garden is a place to sense and to find comfort, to add color and taste to life, and to seek the tranquility of nature. It is a place to feel healthy, optimistic, and alive. The garden is also a place to relate the past and present, bringing personal memories and optimism to everyday life. Twice as many women as men are represented in this meta-ethnography, and the experiences with nature and gardens emerged as being independent of gender. However, the gender perspective did not seem to be an issue in the included studies.

A nursing home has two basic features—a “home” for the older peoples and a place for “nursing.” A garden belongs to a home (Scott et al., 2015), and in nursing homes and residential care gardens, it can be easier for older people to find a place to call home (Johansen & Gonzalez, 2018; Shi et al., 2019). Nursing facilities should endeavor to model themselves accordingly, and the garden can become a symbol of homeliness in a new place (Andrews & Moon, 2005). The idea of home means a safe and secure base that offers protection and rest. Thus, the community surrounding the home is of the utmost importance, but each person needs to have access to their preferred space (Molony, 2010; Tsai et al., 2020). The outdoor space (i.e., the garden) grows in significance as the abilities and movement of the older people get restricted (Raske, 2010). A previous meta-ethnography has confirmed that older people relocating to residential care facilities experience a loss of home (Molony, 2010). Another used the metaphor “Russian babushka doll,” signifying the loss of space and freedom, both physically and cognitively, for those living with dementia (Førsund et al., 2018, p. 22). The garden might have been an important part of their life that they cherished dearly. Also, they might deem it as a substitute for a previously active outdoor life (Johansen & Gonzalez, 2018; Reynolds, 2016). It turns out that older people living in nursing homes often have little contact with nature, even though the garden is closely located, which may cause suffering from discontentment (Raske, 2010; Tsai et al., 2020).

Regarding the other concept, “nursing,” the present meta-ethnography reveals that many of the residents in nursing homes and residential care are depending on help to get and move around outdoors, so nurses play an important role in facilitating the use of the garden (Dahlkvist et al., 2020; Lo et al., 2019). From the residents’ perspective, time is a scarce factor (Dahlkvist et al., 2020), so even though there is easy access to the garden, the residents’ opportunities for outdoor visits are limited. Physical facilitation with, for example, paths with a leveled surface and sitting and resting places may give the residents safe and predictable use of the garden (Berentsen et al., 2008; Winterbottom & Wagenfeld, 2015).

The uniqueness of this study is that the garden emerges as essential to the residents’ health and well-being; therefore, it should have been a natural and integral part of care in nursing homes and residential care (Whear et al., 2014). To address this gap, there is a need for change that includes attitude, knowledge, and awareness (Cooley et al., 2020) to safeguard residents’ relationships with nature and gardens (Andrews, 2006). The association with nature is seen as one of several health sources due to its “reparative processes” (Nightingale, 1860, p. 6). According to Andrews & Moon (2005), placelessness is harmful to the individual’s health. It is noteworthy that the place and environment of care maintain the older peoples’ relationship to nature since nature has a deeper meaning, with cultural anchoring for humans than just being a geographical place (Andrews, 2006). A garden might be such a place.

Closeness and contact with nature and people may help residents live life their way. Besides, being in the garden is frequently associated with a sense of being alive and being respected for originality (Johansen & Gonzalez, 2018; Raske, 2010; Reynolds, 2016). The residents’ past, with good memories about nature, places, and people, is like soil that sustains their well-being in a nursing home (Johansen & Gonzalez, 2018; Reynolds, 2016; Tsai et al., 2020), and in some way, the memories are the glue that holds their lives together.

The garden contributed to significant and eventful days and enabled the residents to reveal their heritage (Dahlkvist et al., 2020; Lo et al., 2019; Tsai et al., 2020), allowing the integration of future garden plans (Reynolds, 2016) into the nursing home environment. In this way, the older people’s habits, life histories, and relations to nature are taken care of and applied in a meaningful way (Andrews & Moon, 2005; Sion et al., 2020). Previous meta-ethnographies show the loss of meaning and loneliness experienced by the older people living in nursing homes and engaging in meaningless activities (Vaismoradi et al., 2016). Another meta-ethnography regarding nursing homes used the metaphor “feeling trapped in an empty waiting room” (Kitzmüller et al., 2018, p. 222), indicating the apathy in a closed environment.

About residents’ usability and access to utilities, little was discussed in the included studies (refer to Table 2), as mentioned by Dahlkvist et al. (2020) and Reynolds (2016). The residents often need help to enter the garden and will also require help to select a preferred activity or place to be alone or with accompaniment. Besides, they would often need help from visiting family and nurses as well. This strategy was, however, seldom witnessed in the present meta-ethnography.

Older people’s wishes and needs related to the maintenance and development of gardens in nursing homes should be on their terms. The cardinality of nature for the residents’ health appeared to be little emphasized and included in nursing, and nurses who are aware of the dependence on nature were better able to cater to the residents’ needs (Magnussen et al., 2019).

The garden became a focal point for activities and recollections (Dahlkvist et al., 2020; Lo et al., 2019) as well as silence (Raske, 2010; Tsai et al., 2020), which promoted sensory health for the body and soul (Lo et al., 2019). Free access to gardens and nature is crucial for older people (Bell et al., 2015; Liao et al., 2020), and when it is inhibited, the sensuousness seems to be deterred, and feelings of unworthiness and suffering, and perhaps death (i.e., at least emotionally), might emerge (Eriksson, 2002). Such an ordeal is not in line with the spirit of Florence Nightingale (1860) and her views.

Using sensory gardens consciously in the care for patients with dementia has proven to create close and appreciative patient-nurse relationships and opens up new domains for nursing care practice (Magnussen et al., 2019). Accordingly, such relationships may form a meaningful context of care that is derived from the ethos of love, responsibility, and sacrifice (Eriksson, 2002).

Routines and rules in the nursing home may create an invisible wall that impedes the use of the garden, especially when it is not included in the routines (Johansen & Gonzalez, 2018; Raske, 2010; Reynolds, 2016; Tsai et al., 2020). The dependence on the garden and similar experiences need to become a “routine” part of nursing care. Nevertheless, its use should also be individualized, and the nursing care plan should account for previous life experiences with outdoor activities and gardens and their meaning. Likewise, nursing care should be based on the older people’s perspectives and should be visible in the present meta-ethnography, encompassing the past, the present, and the future. The human being does not feel whole and well if access is never or too seldom provided to outdoor spaces. Human dignity implies being whole as a human being, and human beings need nature experiences to feel whole and well (Eriksson, 2002). The way of expressing the older peoples’ dignity by nature may vary and needs to be known by the nurse (Magnussen et al., 2019). During the COVID-19, this notion became even more evident.

The older people, regardless of gender, felt gratitude for the garden, as it brought a spark of life into their lives (Dahlkvist et al., 2020; Johansen & Gonzalez, 2018; Lo et al., 2019; Raske, 2010; Reynolds, 2016; Tsai et al., 2020). The garden seems to nourish and strengthen their personality and integrity, and they felt that life was worth living. This nourishment may show some of the symbiotic human-nature relationships that are mentioned by Reynolds (2016) and Tsai et al. (2020). Based on this belief, the outdoor environment in nursing homes must be taken into account if its potential is to be utilized in improving the lives of residents (Bengtsson & Carlsson, 2013).

With regards to the culture of a nursing home, Eriksson (2002) holds the opinion that people who co-create provide a meaningful culture that is inviting, open, and dignified. Hence, a holistic understanding of health, including nature, needs to be harnessed (Eriksson, 2002). The concept of a “human being” includes the relation to oneself, to others, and nature, these relationships facilitating therapeutic processes, such as reducing stress from psychosocial disorders (Corazon et al., 2019) and isolation among refugees (Poulsen et al., 2020). When nature and gardens have such a health-promoting influence on human beings of all ages, we find it necessary to bring a new nature approach into nursing that includes gardens, and we should let the resident’s voice guide individual care.

### Methodological Considerations

The included studies in this meta-ethnography represented older people from different residential care facilities, which might confirm the search strategy. All the included studies were evaluated using the CASP (http://www.casp-uk.net/checklists), and in this process confidentiality and voluntariness were quality assured, as well as the credibility and applicability of the findings. Meta-ethnography is considered a useful method when examining participants’ experiences and perspectives in previous qualitative research. In this study, we followed the eMERGe reporting guidance (France et al., 2019), which contributes to improving the transparency and quality of this meta-ethnography, and our results may serve as robust evidence. The eMERGe guidelines worked as a reminder for clarity of reporting our meta-ethnography (France et al., 2019).

**Figure 3.**
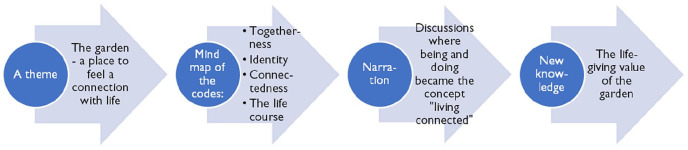
An example of development of new knowledge.

This meta-ethnography provides a reflective, systematic, data-driven synthesis based on literature spanning ten years and reported from six different countries. We included older people’s perspectives of the garden in their nursing home or residential care, and the study thus describes the most important perspective (i.e., the person in care). A qualitative meta-synthesis is always kept separate from a person’s life. Rather than simply relying on retelling, the narration of experiences according to the primary researcher’s descriptions and interpretations results in new knowledge, as illustrated in Figure 3 (Noblit & Hare, 1988).

In this meta-ethnography, we opted for residential care facilities with gardens (Bondas & Hall, 2007). Although gardens are described in different terms, they all have the same purpose, users, and content. Moreover, the studies represent different types of gardens distributed across several countries. Therefore, we decided to exclude studies concerning natural experiences or common public gardens, as these are not planned for older people’s therapeutic use. Data from the studies included were re-analyzed; thereafter, the data were compared and translated, and the end product represented an integration of research findings beyond the usual dependence on aggregation (Noblit & Hare, 1988). The number of included studies, some methodological weaknesses, and the lack of description concerning the facilitation of mobility aids (Table 1) may have affected the basis for the translation and constitute a limitation.

The synthesis process allowed us to reflect and create concepts and themes that shed light on the research findings in a new way, actualizing and enriching the discourse regarding gardens in nursing care (Noblit & Hare, 1988). To generate and provide a theoretical understanding of how this phenomenon is connected and interacts is seen as a strength in meta-ethnography (Bondas & Hall, 2007; Noblit & Hare, 1988). In particular, this meta-ethnography clarifies differences and similarities consistently and fruitfully. “A meta-ethnography is complete when we understand the meaning of the synthesis to our own lives and the lives of others” (Noblit & Hare, 1988, p. 62). Our intention was to illuminate the older persons’ perspectives and thus only the reader can decide if this was accomplished in our study, in order to be of use when developing nursing care and for further research. Finally, we have validated that meta-ethnography brings forth new insights that enlarge understanding and suggest that gardens should be included in nursing care at residential care facilities.

## Conclusion

The overarching metaphor “Human flourishing with dignity” may lead the way for new nature-oriented approaches in nursing care—from the older peoples’ perspective. This meta-ethnography denotes that older people in nursing homes and residential care need contact with nature and often will need assistance to achieve it. Simultaneously, it seems that nature and gardens have not yet fully become an incorporated part of nursing care and, thus, fall short to meet the residents’ needs and longings for contact with nature. The old peoples’ own nature-oriented experiences and the impact on health, well-being, and dignity can be vital contributions to improve care in nursing homes and residential care facilities.

Apparently, nature is embodied within older people, and such experiences must receive greater attention in nursing care. The uniqueness of this study is that the garden, as an integral part of nursing, is essential to health, as per the residents’ perspectives. The health of the residents cannot be fully measured, but from the perspective of the older people, it can be experienced when nature becomes part of their life and the care process. Gaining knowledge from the residents’ perspectives is crucial for developing health care services and health policies for the future, and this mechanism may prove to be ideal in ensuring that older people flourish in nursing care and residential care settings.

### Relevance to clinical practice

Gardens, as an integral part of nursing, are underrated and must be incorporated more actively in practice and education. The garden turns out to be important for older people’s mental and physical health and social fellowship, and it requires more attention than it has received so far. Based on the older people’s perspective, the knowledge about the value of nature for their health and well-being should be increased both in training and practice. In this way, caregivers in nursing homes and residential care might integrate gardens as an essential part of nursing and safeguard the dignity of the individual residents. Integrating gardens as a natural and vital part of nursing requires gardens to become mandatory in nursing education and health policies.

### What does this paper contribute to the wider global clinical community?

This paper contributes new knowledge about the garden’s impact on health, well-being, and human dignity from the resident’s perspective. It is necessary to raise awareness about the garden as a caring and life-giving space important for human dignity. The resident’s voice and preferences for garden use must guide individual nursing care. Gardens are an essential part of nursing for older people residing in nursing homes and residential care facilities; hence, gardens must be a primary part of nursing.
